# Assessment of the Ageing and Durability of Polymers

**DOI:** 10.3390/polym14101934

**Published:** 2022-05-10

**Authors:** Mariaenrica Frigione

**Affiliations:** Innovation Engineering Department, University of Salento, Prov. le Lecce-Monteroni, 73100 Lecce, Italy; mariaenrica.frigione@unisalento.it

As any other natural or industrial material, polymers can experience some kind of degradation during their service life, resulting in minor to severe changes in their properties. The “durability” of a polymeric material, intended as the average life in service conditions, depends on several parameters, the most important being the chemical nature and composition of the polymer, the process used to manufacture and/or to apply it, the usage and load regimes, and the kind and level of environmental exposure.

Thanks to a wide availability of polymeric materials (based on thermoplastic, semi-crystalline and amorphous, cross-linked, thermosetting, elastomer, natural and biodegradable polymers, composites and nano-composites with a polymeric matrix, etc.), these materials are successfully employed in numerous applications and uses. Consequently, they are subjected to different exposure conditions characterizing their application and use. It is, therefore, necessary to study their durability and their long-term performance in the specific service conditions, in order to guarantee their continuous reliability in common as well as in high tech applications. The valuable contributions in the Special Issue “Assessment of the Ageing and Durability of Polymers” provided a unique collection of original studies aimed at evaluating the durability of polymeric materials applied in widely different fields. In their investigations, the contributors often proposed innovative solutions in terms of new techniques and methodologies to study the durability of the polymers under particular environmental conditions. Aside from this, new formulations or additives were also experimented with the aim to improve the resistance of the polymeric materials against different aging regimes. On the other hand, every new material, before being accepted by the market, must be analyzed and validated to predict its long-term behavior and performance. Criticisms relating to the methods and procedures available to predict the long-term performance of polymeric materials were, finally, highlighted, providing useful suggestions for further study in this field.

The durability of cross-linked polyethylene (XLPE) employed in cables for electric applications was analyzed in [1], relating the modifications induced by gamma radiations to the composition of the polymer. Materials for cables must respect strict safety requirements, especially when used in nuclear or electrical power plants. The authors of the investigation found that the effects of the radiative ageing on cables appreciably depends on the absorbed dose amount and rate, the lower the dose rate, the greater the degradation. However, by changing the formulation of the polymeric material composing the cables, it is possible to mitigate the effects of radiative ageing. Among the different experimented additives, the study proved that the addition of an antioxidant to cross-linked polyethylene improves the cable durability, avoiding the degradation of the polymer at any dose and any dosage rate. Moreover, a proper combination of different additives could be effective in protecting the polymeric material composing the cable from multiple degradation factors.

The ageing of PVC films due to UV radiation exposure was the subject of the studies illustrated in [2,3], whose results can be taken as a reference for outdoor applications. The aim of the first manuscript [2] was to verify whether the addition of new tin complexes containing atenolol is effective in improving the durability to radiations of PVC films. The authors of this study found that atenolol-tin complexes, in particular the triphenyl-tin complex, due to its high aromaticity, provide significant photo-stabilization to the PVC films, as verified by infrared spectroscopic (FTIR) analysis and weight loss measurements. The tin complex additives were, in fact, able to act as effective light absorbers, hydrogen chloride scavengers and hydroperoxide decomposers. An improved resistance to photo-degradation of the PVC films was obtained also with the addition of different organotin complexes, as described in [3]. It was confirmed that an aromatic nature of organotin complexes offered a greater capacity in inhibiting the photo-degradation of PVC films, absorbing UV light and scavenging radicals, hydrogen chloride and peroxides.

The importance of analyzing the effects of UV radiations on polymers performance is also evidenced by the experimental work described in [4]. The polymer object of this investigation was a silicone rubber, which was exposed for different times to UVA (at 340 nm) and UVB (at 313 nm) lamps. Low molecular weight siloxanes, extracted by a soxhlet extraction process after the UV ageing procedure, were analyzed by gel chromatography technique, demonstrating that silicone rubber experienced two different processes if irradiated by UVA/UVB radiations: UVB radiation was responsible for the degradation through chain scission in silicone rubber; on the other hand, UVA was likely to cause a post-cure in the rubber structure, completing the cross-linking of the cyclic oligomers. The different effects of the diverse UV radiations were attributed to the higher photon energy of UVB compared to that of UVA.

The effect of the composition of commercial polypropylenes for pipes on the polymers durability was the subject of the paper [5]. Taking into account the typical conditions experienced by a duct, which must carry hot and cold water in the presence of high pressures, the aging was performed in hot (110 °C) water, subjecting the pipes to hydrostatic pressure. The degradation of the polypropylenes under investigation was manifested in a shift of the melting point of the polymers to higher temperatures, with an increase in the degree of crystallization, both phenomena probably due to a degradation of the amorphous fraction of the polymer. Furthermore, the aging provoked also a complete or substantial loss of additives, mainly antioxidants. However, the presence of a layer of glass fibers allowed the pipe to withstand pressure and high temperature, slowing down the propagation of fractures caused by the degradation. The highest durability was offered by the polypropylene containing the greatest number of antioxidants, with the addition of the additive TiO_2_. The authors of the study concluded that polypropylene pipes of adequate composition and containing an appropriate mix of antioxidants can well tolerate severe aging caused by the service conditions.

The durability assessment of epoxy adhesives, often used in structural applications, is essential to guaranteeing their reliability, especially in demanding applications. In this regard, the effect of the exposure to acidic environments over different time spans on the mechanical properties of two epoxy resins containing calcium carbonate (CaCO_3_) micro-particles was analyzed and presented in [6]. It was found that the exposure to acidic environments negatively influenced the compressive characteristics of the epoxy compounds, the higher the pH of the solution and the longer the immersion time, the greater the reduction in mechanical properties. The addition of the calcium carbonate filler showed no particular advantages in terms of improvements in the durability to this aging factor of epoxy resins.

A wide literature survey on the durability and long-term performance of fiber-reinforced polymer (FRP) composites, based on epoxy resins and employed for the reinforcement or rehabilitation of concrete structures, is presented in [7]. External bonded FRPs represent, in fact, an advantageous and effective solution to strengthen and extend the service life of aged concrete constructions. The success of this technique, on the other hand, is limited by a poor knowledge of the durability of FRPs, especially when exposed to harsh environmental conditions. The paper illustrated the factors affecting the durability of FRPs, from the selection of the materials to the intervention design, from the application techniques to the type of exposure. It also described how the different factors can interact, giving rise to degradation mechanisms involving the epoxy matrix, the fibers and the interfaces among epoxy and fibers and among epoxy and concrete. A section of the paper was devoted to the available international guidelines for designing with FRPs, highlighting how they could be improved to more realistically take into account the durability of such materials. Finally, new nano-modified epoxy formulations were reviewed as materials potentially capable of developing FRPs with improved durability, even in harsh environments.

Equally current and important is the study of the durability of new materials and technologies used for the protection and conservation of ancient constructions and cultural heritage sites, which deserve to be preserved as they represent precious treasures of the culture and traditions of the past. In this context, the improvements in the self-cleaning ability and durability of Si-based consolidants for granite slabs, due to the addition of different amounts of nanocrystalline TiO_2_, activated by UVA or UVB radiations, was analyzed in [8]. The authors of this study demonstrated that the self-cleaning efficacy is a function of the composition of the consolidant: the self- efficacy was, in fact, improved at greater TiO_2_ contents in the silica-based consolidants. It was affected also by UV radiations, greater pollutant degradation rates were, indeed, achieved upon exposure to UVB radiations. It was found, finally, that the natural color of the stone was almost completely restored as a consequence of the degradation of the pollutants on its surface.

In the field of polymeric materials for cultural and architectural heritage conservation, fluoropolymers are widely used as protective coatings due to a favorable combination of mechanical characteristics, chemical resistance and high transparency. Once these products are applied on outdoor exposed surfaces, to improve their resistance to weathering, the coatings themselves can be degraded by external agents. Therefore, it is necessary to study their durability in different environments to be sure that their protective action will be not negatively affected. The experimental work described in [9] tried to address this issue, analyzing two commercial fluropolymers, namely perfluoro alkoxy (PFA) and ethylene tetrafluoroethylene copolymer (ETFE), subjected to an accelerating aging procedure. This latter consisted in repeated cycles composed by a 6 h exposure to UVA radiations, followed by 3 h of water condensation, with the aim of reproducing the environmental conditions occurring in the city of Rome. The mechanical characteristics and chemical–physical properties of the fluorinated polymer films were evaluated before and after the exposure to the accelerating aging, in order to assess the environmental resistance of both fluropolymers. The results of the performed tests allowed to conclude that ETFE copolymer offers the best performance, both before and after the accelerated aging.

The reliability of accelerated aging procedures, proposed to predict in short times the durability of a polymer long exposed to different external agents, is a highly debated topic, as extensively illustrated in [10]. In such procedures, the polymers are exposed to aging regimes much more severe than those that they would experience in true outdoor conditions (high temperatures and hot water, massive levels of radiations), with the aim of accelerating the degradation kinetics of polymers reducing the times for analysis. The paper underlined that the extreme aging conditions employed in these tests can trigger in polymers reactions and degradation processes that would not take place in real conditions, collecting unrealistic results. Furthermore, to supply representative data, the acceleration factors should take into account the different environmental conditions characteristic of the geographical areas of exposure they want to reproduce; this would imply the identification of different standards for each climatic zone. A review of the available standard codes to perform accelerated aging tests on polymers was presented by the authors, evidencing a general lack of indications on how to correlate the results of the accelerated aging tests for each different class of polymers with those obtained on naturally exposed materials. The paper, finally, illustrates some issues on which research should be primarily focused in order to make these methodologies more functional.

The theme of the different effects caused on polymeric materials by natural aging, relating to different geographical areas, is also the topic addressed in the experimental work presented in [11]. The degradation of pristine and stabilized polycarbonate specimens exposed during two years in five different locations, in Japan and the US, was monitored with the assessment of changes in color and gloss, recording at the same time the climate variations. The contribution of each single aging factor (temperature, moisture, UV radiations) on the property modifications measured on polymers was evaluated, correlating this information with the natural climatic conditions observed in the different regions. On the basis of their results, the authors proposed a model to predict the degradation rate of the polymers as a function of the environmental parameters characteristic of each climatic area.

The prevision of the long-term performance of acrylonitrile sealing elements employed in nuclear plants is the subject of the research presented in [12]. The suggested approach implied the measurement of Shore A hardness on the acrylonitrile components after their exposure to different temperatures for diverse ranges of time, correlating the exposure conditions to the hardening observed in the polymer, the latter having a direct correlation with the durability in service. From these evaluations, the authors were able to estimate the time after which the acrylonitrile sealing elements lose reliability and must be replaced. The advantage of the proposed method highlighted in the article laid in the use of a non-destructive and easily performed test as an effective tool for the safe maintenance and management of safety devices.

The analysis of the effect of the application conditions on the performance of an adhesive was the subject of the study reported in [13]. The quality of the bond developed by a divinylsiloxane-bis-benzocyclobutene (DVS-BCB) adhesive was, in fact, related to the presence of voids, caused by an inadequate compression pressure and to an unsatisfactory cross-linking density depending on the processing temperature. The studies, performed with the aid of spectroscopic techniques for the measurement of the cross-linking density and mechanical tests, to evaluate the adhesion strength, allowed the identification of the best conditions in terms of processing temperature and applied pressure, which guarantee a good void-free bonding. The optimization of the process conditions illustrated in the paper could find immediate application in optical and electronic devices.

## Data Availability

Not applicable.
